# Does the Size of Cervical Disc Herniation Affect Clinical Parameters in Cervical Radiculopathy?

**DOI:** 10.3390/jcm14248900

**Published:** 2025-12-16

**Authors:** Azra Gül, Xiaoyu Yang, Caroline M. W. Goedmakers, Carmen Vleggeert-Lankamp

**Affiliations:** 1 Department of Neurosurgery, Leiden University Medical Centre, 2333 ZA Leiden, The Netherlandsc.l.a.vleggeert-lankamp@lumc.nl (C.V.-L.); 2 Department of Neurosurgery, Computational Neuroscience Outcomes Center at Harvard, Brigham and Women’s Hospital, 75 Francis St, Boston, MA 02115, USA; 3 Department of Neurosurgery, Boston Medical Center, Boston, MA 02118, USA; 4 Department of Neurosurgery, Spaarne Gasthuis, 2134 TM Haarlem/2035 RC Hoofddorp, The Netherlands

**Keywords:** cervical radiculopathy, ACDF, conservative management, VAS arm pain, inflammation, herniation size

## Abstract

**Background/Objectives:** The extent to which compression or inflammation contribute to the development of cervical radiculopathy and its associated symptoms remains unclear. This study aimed to evaluate whether herniated disc size correlates with baseline symptom severity and/or clinical outcome in patients with cervical radiculopathy, treated surgically or conservatively. **Methods:** This multi-centre retrospective cohort study included 206 patients with cervical radiculopathy due to a herniated disc. MRI scans from 108 patients in the NECK trial (surgical treatment, evaluating disc replacement) and 98 from the CASINO trial (surgical versus conservative treatment), were used to classify herniation size. Clinical outcome was assessed using the Visual Analogue Scale for arm and neck pain, the Neck Disability Index and the EuroQol VAS at baseline and one year after treatment. Binary logistic regression models were used to evaluate associations between herniation size and clinical outcome, adjusting for treatment type, gender, Body Mass Index and age. **Results:** A total of 107 patients presented with a small herniation, and 99 had a large herniation. Baseline mean NDI scores were 39.1 ± 15 (surgical group, *n* = 165) and 32.9 ± 16 (conservative group, *n* = 41). No association was observed between herniation size and clinical condition (OR 1.010, *p* = 0.323). After one year, mean NDI scores were 16.2 ± 15 (surgical group) versus 19.6 ± 22 (conservative group), with no significant associations between herniation size and outcome in either group. Similar findings were found for VAS arm and neck pain and EQ-VAS. **Conclusions:** Disc herniation size on baseline MRI showed no correlation with symptom severity or one-year clinical outcomes, suggesting that inflammatory mechanisms, rather than mechanical compression alone, play a key role in cervical radiculopathy.

## 1. Introduction

A herniated cervical disc (cHNP) is typically the underlying cause of cervical radiculopathy, a neurological disorder that arises from compression and/or irritation of a cervical nerve root in the spine. The incidence of cHNP is described as 1.79 per 1000 individuals, with an approximated prevalence of 5.8 per 1000 individuals [1]. Patients often experience pain that radiates into the arm along a radicular pattern, frequently accompanied by neck pain. Cervical radiculopathy is regularly attributed to the compression of the nerve root by a bulging or herniated disc [2]. However, cervical radiculopathy may also result from inflammation of the nerve root or its surroundings [3]. The extent to which compression or inflammation contributes to the development of cervical radiculopathy and its associated symptoms remains unclear.

Types of cervical disc herniations are classified by imaging and clinical features into protrusion, extrusion, and sequestration, each with varying potential to compress the nerve root [4]. The majority of patients with cHNP experience substantial improvement with conservative management within the first 6 months after onset, receiving a combination of physical therapy, oral analgesics, and patient education [5]. However, when symptoms do not resolve adequately, patients are often referred for surgical management, commonly involving an anterior discectomy. Although surgery may yield faster and more pronounced pain relief in select patients, long-term outcomes do not consistently favour one approach over another. Therefore, the choice of intervention should be individualised based on clinical presentation, patient preference, and surgeon expertise [6,7].

Magnetic Resonance Imaging (MRI) is the diagnostic modality of choice in patients with suspected cervical disc herniation and is widely employed in both diagnostic evaluation and treatment planning. It provides a non-invasive, high-resolution assessment of cervical spinal anatomy, revealing degenerative changes and enabling the identification and measurement of disc herniation size and shape, as well as the dimensions of the spinal canal, thereby helping to clarify the cause of clinically observed cervical radiculopathy [8].

Previous research has investigated the impact of treatment modalities for cervical disc herniation in two prospective trials: the NECK trial [9] and the CASINO trial [10]. The NECK trial evaluated anterior discectomy comparing three distinct intervertebral devices, whereas the CASINO trial compared conservative versus surgical management in patients with cervical radiculopathy. At baseline and at one year after treatment, no statistically significant differences in clinical parameters could be substantiated between the patients’ groups evaluated in the NECK and in the CASINO trial. In all patients, either being treated surgically or conservatively, the baseline clinical parameters were comparable, and, likewise, one year after treatment, the clinical parameters in all patients were in the same range. Based on these findings, the current study merges the populations from both trials to explore the relationship between the size of cervical disc herniations and the experienced clinical symptoms. In addition, we seek to examine whether the size of a disc herniation influences the effectiveness of surgical or conservative treatment.

## 2. Materials and Methods

### 2.1. Study Design

This article utilised data from both the CASINO Trial and the NECK Trial: The CASINO trial (NTR3504) [10] has been conducted as a prospective, multi-centre observational cohort study endeavouring to compare outcomes of patients with cervical radiculopathy caused by intervertebral disc herniation after conservative or surgical treatment. After presentation to the neurosurgeon, conservative and surgical treatment were discussed, and once all were in agreement, a decision was made. The Netherlands Cervical Kinetics trial (NECK trial; NTR1289) [9] is a prospective, randomised, double-blind multi-centre study, designed to evaluate and compare outcomes among three treatment groups: anterior cervical discectomy without intervertebral device (ACD), with an intervertebral PEEK cage (ACDF, cage standalone), and anterior cervical discectomy with arthroplasty (ACDA; activC^®^). Both trials are intended to refine treatment strategies and enhance patient care and are in accordance with the World Medical Association Declaration of Helsinki.

### 2.2. Patient Characteristics

The CASINO trial included patients aged 18 to 75 years who were diagnosed with cervical radiculopathy syndrome (CRS) due to a herniated disc. Eligible patients had to experience at least two months of disabling symptoms or tingling sensations. Additionally, either the VAS arm pain or the VAS tingling sensations should be ≥40 mm. Patients with arm paresis (MRC < 4), predominant myelopathy symptoms, previous cervical surgery, or instability requiring instrumented spondylodesis were excluded. Other exclusion factors were pregnancy, insufficient knowledge of the Dutch language, planned emigration, or participation in another clinical trial. After presentation to the neurosurgeon, conservative and surgical treatment were discussed, and, once all were in agreement, a decision was made. In the CASINO trial, 141 patients were enrolled, of whom 53 patients were treated conservatively, and 88 patients underwent surgical anterior decompression with implantation of a PEEK cage. Baseline MRI data were available for 98 patients.

The NECK trial likewise included patients aged 18 to 65 years with CSR due to a herniated disc, who had received more than eight weeks of conservative treatment. Exclusion criteria included prior cervical spine surgery, absence of pathological (>2 mm) motion or spondylolisthesis at the target level, narrow disc levels (<3 mm), kyphosis, predominant myelopathy, metabolic or bone diseases, insufficient Dutch language skills, or planned emigration within a year. In the NECK trial, 111 patients were recruited and randomised into three treatment groups: 35 patients underwent ACDA, 38 patients underwent ACDF, and 38 patients were treated with ACD. MRI data were obtained from 108 patients at the time of enrolment.

Both studies received ethical approval from the Medical Ethics Review Committee in the Netherlands, and written informed consent was obtained from all patients prior to inclusion. The design and protocol of the NECK trial have been previously published [7] as well as the 2- and 5-year outcome data [9,11].

### 2.3. Outcome

To assess clinical outcome at baseline and 1-year follow-up, four different measures were employed. The Neck Disability Index (NDI) served as the primary outcome measure. Secondary outcome measures involved arm pain, neck pain and patient satisfaction. Arm and neck pain were evaluated using the Visual Analogue Scale (VAS), and patient satisfaction was measured with the EuroQol VAS (EQ-VAS).

The NDI is a questionnaire consisting of 10 items that assess neck functionality by three distinct aspects: pain intensity, daily work-related activities and non-work-related activities. Each item is scored on a scale from 0 to 5, resulting in a total score ranging from 0 (best score) to 50 (worst score). The 50-point score is first converted to a 100 percent score and then into a 0–100 scale. The NDI is derived from the Oswestry Low Back Pain Index and is recognised for its reliability and validity in evaluating patients with cervical pathology [12,13,14].

Pain severity in the arm and neck was assessed by using the VAS. The VAS arm score illustrates the disabling pain/tingling intensity in the arm, with a score ranging from 0 to 100. Further, 0 mm means ‘no pain or tingling sensations’ and 100 mm means ‘the most terrible pain or tingling sensations I can imagine’. Reliability, validity and responsiveness of the VAS have been demonstrated previously [15,16]. The VAS neck score was scored likewise.

Lastly, the EQ-VAS was used to assess a patient’s self-perceived health. It ranges from 0 to 100 points, where 0 stands for ‘worst imaginable health’, and 100 represents ‘best imaginable health’.

### 2.4. MRI Evaluation

**Figure 1:**
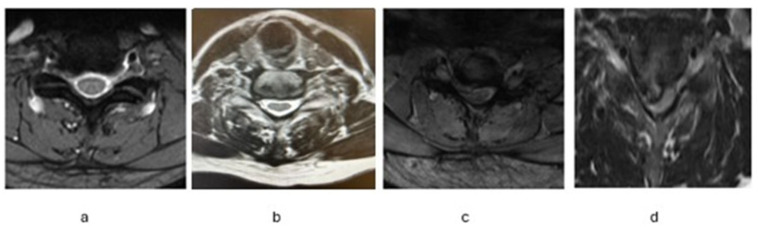
Classification of Cervical Disc Herniation Size: (**a**) slight bulging, (**b**) small Herniated disc, (**c**) moderate herniation, and (**d**) severe Herniation.

MRI scans were conducted at baseline using a protocol designed for 1.5 or 3 Tesla machines. The imaging included standard sagittal T1 and T2 sequences, along with axial T2 images, acquired with 3 mm contiguous slices and an in-plane resolution of 1 mm^2^ or finer. Disc herniation size was assessed at the affected side and graded as follows: slight bulging, small herniated disc, moderate herniation pro/extrusion with less than ¼ of the canal and severe herniation with pro/extrusion more than ¼ of the canal (Figure 1). All cervical herniations were paramedian, as foraminal cervical herniations typically are treated posteriorly, and median cervical herniations typically give complaints of myelopathy. The MRIs were evaluated by neurosurgeons in the participating hospitals and additionally independently re-evaluated by a senior neurosurgeon (CVL), all blinded to clinical outcomes. Prior to the start of this study, participating neurosurgeons underwent a standardized training session using test cases to ensure uniform application of the grading criteria. In cases of disagreement, the final classification was based on the assessment of the senior neurosurgeon. To assess the reliability of the grading system, a subset of MRI scans was re-scored to evaluate both inter- and intra-rater agreement, yielding satisfactory consistency (Appendix A).

### 2.5. Statistical Analysis

Normally distributed variables are presented as mean ± standard deviation (SD). Binary logistic regression models have been performed to compare the odds between large versus small herniation size on having a higher and thus worse clinical outcome, at baseline and at 1-year follow-up. Additionally, separate analyses of the patients from the NECK trial, the patients in the CASINO trial who received surgical treatment and the patients in the CASINO trial who received conservative treatment were performed. All the analyses were adjusted for baseline values, gender, age and Body Mass Index (BMI). The overall analysis was also adjusted for treatment type: surgical or conservative. A *p*-value of 0.05 or lower was considered statistically significant.

Data collection and checking for quality were performed with the ProMISe data management system of the Department of Medical Statistics and BioInformatics of the Leiden University Medical Centre. IBM SPSS software, version 30.0, was utilised for all statistical analyses.

## 3. Results

### 3.1. Herniation Size

**Figure 2:**
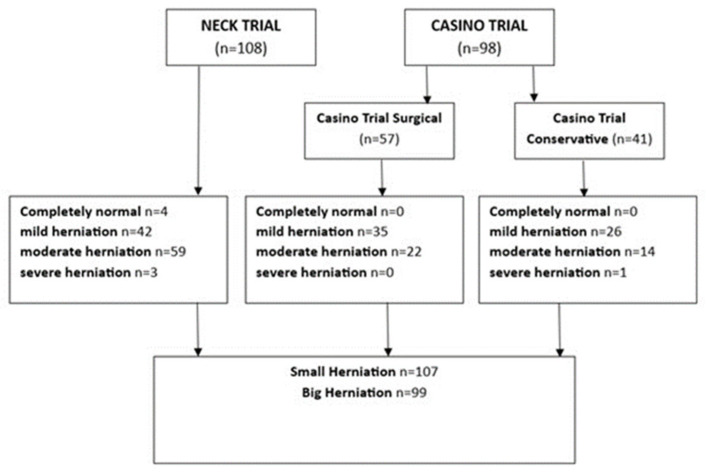
Patient Distribution.

A total of 206 patients were eligible for inclusion, of whom 98 were from the CASINO Trial, and 108 were from the NECK Trial. In both the NECK and the CASINO trial, the number of patients demonstrating slight bulging was very limited (Figure 2). Likewise, the number of patients demonstrating a severe size of herniation was very small. Therefore, the data were dichotomised by combining ‘slight bulging’ and ‘small herniated disc’ into small disc herniation (*n* = 107) and combining ‘moderate herniated disc’ and ‘severe herniated disc’ into ‘large disc herniation’ (*n* = 99). This dichotomisation was chosen to ensure sufficient sample sizes in each group for meaningful statistical analysis.

### 3.2. Clinical Parameters

At baseline, the small herniation group and the large herniation group showed comparable demographic and clinical characteristics (Table 1). In the small herniation group, the mean age was 49.8 ± 9.7 and 48.0 ± 9.0 in the large herniation group. Smoking prevalence, BMI, VAS arm pain, VAS neck pain, NDI and EQ-VAS were comparable between the two groups.

**Figure 3:**
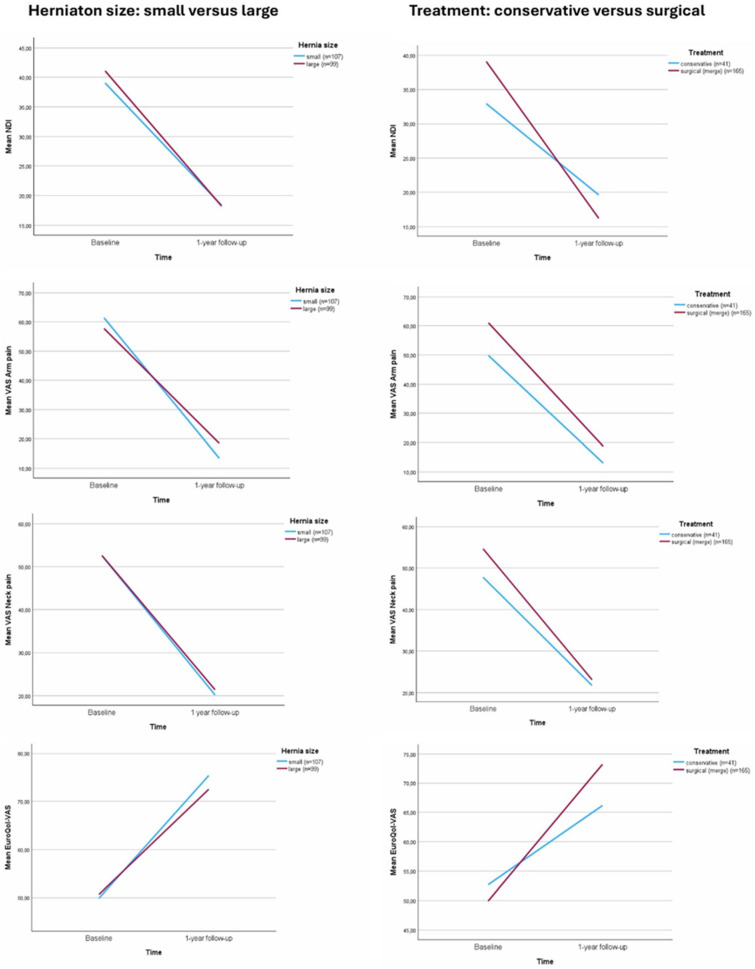
Clinical outcomes at baseline and 1 year of follow-up: Neck Disability Index (NDI), Visual Analogue Scale (VAS). There were no signficant differences when comparing small versus large herniation size.

At baseline, the mean NDI score in the surgically treated patients was 39.1 ± 15 compared to 39.0 ± 16 in the conservatively treated patients (*p* = 0.929), which decreased to 16.2 ± 15 and 19.6 ± 22 after one year of follow-up (*p* = 0.361) (Table 2, Figure 3). The mean VAS arm pain scores were 61.1 ± 23 in the surgical arm during enrolment and 49.8 ± 28 in the conservative arm (*p* = 0.009). After one year, the mean VAS arm pain improved to 18.7 ± 25 in the surgical arm and 13.0 ± 22 in the conservative arm (*p* = 0.197). At baseline, the mean VAS neck pain was 54.6 ± 26 in the surgical treatment group versus 47.8 ± 29 in the conservative treatment group (*p* = 0.119). One year after inclusion, this was 23.1 ± 25 versus 21.7 ± 28 (*p* = 0.778). The mean EQ-VAS increased within both treatment groups: from 49.9 ± 23 versus 52.7 ± 23 at baseline (*p* = 0.438), to 73.2 ± 21 versus 66.2 ± 28 after one year (*p* = 0.139) (Table 2, Figure 3). Linear regression models did not show any significant differences between clinical outcomes at baseline nor after one year in the large versus small herniation size groups (Appendix B).

### 3.3. Associations

Within the surgical treatment arm (merged), the adjusted odds ratio for having a higher (and thus worse) NDI score between patients with large versus small sizes at baseline was 1.007 (*p* = 0.551). In the conservative treatment arm, the adjusted odds ratio was 1.010 (*p* = 0.629) (Table 3). After one year of follow-up, the adjusted odds ratio was 1.001 (*p* = 0.925) in the surgically treated patients and 1.538 (*p* = 0.608) in the conservative treatment group.

No significant difference in the odds of having a higher VAS arm pain score was observed between patients with large versus small herniation sizes at baseline in both the surgical group (OR 0.994, *p* = 0.476) and in the conservative group (OR 0.991, *p* = 0.487). Similarly, after one year of follow-up, the odds remained comparable (OR 1.009, *p* = 0.262 versus OR 1.003, *p* = 0.901). The odds of having a higher VAS neck pain score demonstrated no significant difference at baseline (surgical OR 1.000, *p* = 0.987 versus conservative OR 0.993, *p* = 0.564) and neither after one year (surgical, OR 0.999, *p* = 0.941 versus conservative, OR 1.006, *p* = 0.660). For EQ-VAS, the odds ratio at baseline was 1.003 (*p* = 0.706) in the surgical group and 0.988 (*p* = 0.475) in the conservative group, remaining similar after one year (OR 0.986, *p* = 0.128 vs. OR 0.975, *p* = 0.630) (Table 3).

## 4. Discussion

No significant association could be established between the size of cervical disc herniation and any of the clinical parameters related to cervical radiculopathy. Neither was the size of hernia predictive of the clinical outcome one year later, regardless of whether the patient was treated surgically or conservatively.

In previously conducted research by Yang et al. [17], similar findings to the current study were observed, demonstrating that cervical herniation size in patients with cervical radiculopathy did not correlate with clinical outcome parameters. Yang et al. focused solely on surgically treated patients, whereas our study aimed to additionally evaluate the correlation for conservatively treated patients. Moreover, our study aimed to expand the number of surgically treated patients to increase statistical power. However, despite this broader approach, our results led to the same conclusion. One could argue that the different treatment strategies within the NECK and CASINO trials might play a role and influence the results; however, no significant differences could be substantiated between the treatment arms in both studies during the first year after inclusion. These findings suggest that herniation size alone does not provide a reliable basis for determining whether to proceed with conservative treatment or to choose surgical treatment.

Hypothetically, it is reasonable to expect that patients with larger disc herniations experience more pain and discomfort due to more compression of the spinal nerve. Likewise, it would be reasonable to expect that patients with larger disc herniations would demonstrate superior improvement upon surgery, as the relief of pressure is more abundant with a greater volume of herniated tissue. Conversely, in cases of a bulging disc, where nerve compression is often less pronounced, poorer recovery outcomes or a wider variability in treatment response might be anticipated. However, our findings do not support these assumptions, as no significant differences in recovery patterns were observed between patients with smaller versus larger herniations.

This finding reinforces the idea that symptom progression may primarily be driven by immunological processes, rather than mechanical compression. Neuroinflammatory mechanisms are increasingly recognised to play a key role in the pathogenesis and the clinical course of radiculopathy. The majority of high-quality studies on neuroinflammation and disc herniation have focused on the lumbar spine, resulting in a relative scarcity of cervical-specific data [18,19,20,21,22]. Nonetheless, it is hypothesised that similar neuroimmune and biomechanical mechanisms underlie both lumbar and cervical radiculopathy [4,23]. Evidence from lumbar disc herniation studies demonstrates that cytokine-mediated neuroimmune activation, glial cell involvement, and central sensitisation contribute to pain and disability independently of mechanical compression [24]. While mechanical factors such as nerve root impingement and canal compromise may influence acute symptoms and surgical outcomes, the lack of consistent correlation between disc herniation size and clinical severity in cervical radiculopathy suggests that inflammatory and neuroimmune processes are equally, if not more, important in driving symptom persistence and recovery [4,25].

Bulging discs and degenerative changes observed on MRI are frequently incidental findings. Nakashima et al. [26] reported that nearly 90% of asymptomatic individuals exhibited cervical disc bulging, defined as posterior disc protrusion exceeding 1 mm. Although studies evaluating healthy individuals remain scarce, these findings raise the possibility that, in some patients presenting with arm pain mimicking CRS, nerve root irritation may not be the primary underlying cause. This could, in part, account for the absence of significant correlations observed in the present analyses, as well as the suboptimal clinical outcomes reported in a subset of patients following surgical intervention. These observations highlight the need for a more comprehensive understanding of the pathophysiology of cervical radiculopathy, particularly with regard to the potential contribution of inflammatory mechanisms.

A limitation of our study is the absence of follow-up MRI at a one-year time point, which precluded assessment of potential changes in herniation size over time. Although the analysis was adjusted for key covariates including age, sex and treatment modality, unmeasured confounding factors may have influenced the results. In particular, psychological factors such as anxiety and depression, known to impact pain perception, could not be accounted for. The Hospital Anxiety and Depression Scale (HADS), which allows for standardised assessment of these variables, was not available for patients enrolled in the CASINO trial, limiting the ability to adjust for their potential effects. Moreover, the inequality of the surgical versus conservatively treated patient groups may introduce bias. Nevertheless, this study remains robust due to its prospective design and well-characterised patient populations, supporting the reliability of our findings. Typical postoperative care after ACDF includes early mobilisation, multimodal pain management, and monitoring for common complications, while formal rehabilitation is not standardised and is often tailored to patient recovery. Although neither trial implemented a structured rehabilitation programme, minor variability in routine postoperative practices across centres cannot be fully excluded.

## 5. Conclusions

The findings of our study emphasise the limited role of cervical disc herniation size in predicting clinical outcomes for patients with cervical radiculopathy. Our results reveal no significant association between herniation size and clinical outcomes, neither after surgical nor conservative treatment. These findings align with previous research, highlighting that disc herniation size is not an important factor in making treatment decisions.

## Figures and Tables

**Table 1:** Patient demographics. Baseline characteristics of all patients in the small and large herniation groups. No statistical differences were presented between the two groups, using an independent t-test for the continuous variables and a chi-squared test for the dichotomous variables.

	Small Herniation *N* = 107	Large Herniation *N* = 99	*p*
Age (years; mean ± SD)	49.8 ± 9.7	48.0 ± 9.0	0.143
Male, *n* (%)	42 (39)	54 (55)	0.077
Smoking, *n* (%)	38 (36)	41 (41)	0.384
Body Mass Index (kg/m^2^; mean ± SD)	26.4 ± 4.2	27.0 ± 4.1	0.200
Baseline VAS Arm pain	61.4 ± 23.2	57.7 ± 24.2	0.149
Baseline EQ-VAS	49.9 ± 23.2	50.7 ± 23.1	0.856
Baseline NDI	44.6 ± 15.2	43.8 ± 16.0	0.750
Baseline VAS Neck Pain	52.5 ± 28.1	52.6 ± 26.4	1.000

Abbreviations: SD, standard deviation; VAS, visual analogue scale; EQ, EuroQol; NDI, neck disability index.

**Table 2:** Clinical outcome at baseline and after one year of follow-up with standard deviations. *p*-values for the between-treatment comparisons are given for each time point comparing the surgical (merge) group versus the conservative patients, calculated using an independent t-test for the continuous data in SPSS.

	Baseline	1-Year Follow-Up
**NDI** NECK trial (Surgical) CASINO trial (Surgical) Surgical (Merge) CASINO trial (Conservative) *p*-value **VAS arm pain** NECK trial (Surgical) CASINO trial (Surgical) Surgical (Merge) CASINO trial (Conservative) *p*-value **VAS neck pain** NECK trial (Surgical) CASINO trial (Surgical) Surgical (merge) CASINO trial (Conservative) *p*-value **EQ-VAS** NECK trial (Surgical) CASINO trial (Surgical) Surgical (merge) CASINO trial (Conservative) *p*-value	44.2 ± 15 37.4 ± 14 39.1 ± 15 32.9 ± 16 0.929 61.2 ± 21 60.9 ± 25 61.1 ± 23 49.8 ± 28 0.009 53.6 ± 26 55.9 ± 26 54.6 ± 26 47.8 ± 29 0.119 48.3 ± 24 51.8 ± 23 49.9 ± 23 52.7 ± 23 0.438	19.0 ± 17 17.3 ± 14 16.2 ± 15 19.6 ± 22 0.361 19.7 ± 26 17.0 ± 23 18.7 ± 25 13.0 ± 22 0.197 23.9 ± 25 21.7 ± 24 23.1 ± 25 21.7 ± 28 0.778 72.7 ± 22 74.1 ± 21 73.2 ± 21 66.2 ± 28 0.139

Abbreviations: NDI, neck disability index; VAS, visual analogue scale; EQ, EuroQol.

**Table 3:** Binary Logistic Regression Models. The odds ratios compare large versus small cervical herniation sizes for having a higher clinical outcome. Within the NDI, VAS arm and VAS neck pain, a higher score means a worse outcome. For the EQ-VAS, a higher score means a better outcome.

		NECK Trial (Surgical)			CASINO Trial (Surgical)			Surgical (Merge)			CASINO (Conservative)			NECK + CASINO		
		Odds	95% CI	*p*	Odds	95% CI	*p*	Odds	95% CI	*p*	Odds	95% CI	*p*	Odds	95% CI	*p*
**NDI**	**baseline**	1.003	0.98; 1.03	0.858	1.000	0.95; 1.05	0.992	1.007	0.98; 1.03	0.551	1.010	0.97; 1.05	0.629	1.010	0.99; 1.03	0.323
	**1 yr FU**	0.999	0.97; 1.02	0.968	0.766	0.14; 4.11	0.756	1.001	0.98; 1.02	0.925	1.538	0.30; 1.08	0.608	1.000	0.98; 1.02	0.989
**Vas arm**	**baseline**	0.990	0.97; 1.0	0.338	1.001	0.98; 1.03	0.959	0.994	0.98; 1.01	0.476	0.991	0.97; 1.02	0.487	0.995	0.98; 1.01	0.405
	**1 yr FU**	1.006	0.99; 1.02	0.511	0.681	0.133; 3.48	0.645	1.009	0.99; 1.02	0.262	1.003	0.96; 1.04	0.901	1.009	0.995; 1.02	0.221
**Vas neck**	**baseline**	0.999	0.98; 1.02	0.890	1.001	0.98; 1.03	0.959	1.000	0.99; 1.01	0.987	0.993	0.97; 1.02	0.564	0.999	0.99; 1.01	0.865
	**1 yr FU**	0.989	0.97; 1.0	0.222	0.681	0.13; 3.48	0.645	0.999	0.99; 1.01	0.941	1.006	0.98; 1.03	0.660	1.001	0.98; 1.01	0.891
**Eq-vas**	**baseline**	1.005	0.99; 1.02	0.586	1.003	0.98; 1.03	0.836	1.003	0.99; 1.02	0.706	0.988	0.96; 1.02	0.475	0.999	0.99; 1.01	0.865
	**1 yr FU**	0.989	0.90; 1.10	0.816	0.556	0.10; 3.23	0.513	0.986	0.97; 1.00	0.128	0.975	0.88; 1.08	0.630	1.001	0.99; 1.01	0.891

Adjusted for Age, Sex, Body Mass Index.

## Data Availability

The data presented in this study are available on request from the corresponding author; hereafter, an application must be submitted to the Medical Ethical Committees of Leiden University Medical Centre.

## Appendix A. Inter- and Intra-Rater Reliability of MRI-Based Herniation Classification

A subset of 41 MRI scans (20%) was random selected for the reliability analyses. Inter-rater reliability was assessed by having two trained neurosurgeons independently grade 41 scans. Intra-rater reliability was assessed by having one neurosurgeon in the participating hospitals re-grade the same 41 scans after 1 month. The inter- and intra-rater agreement was evaluated using weighted Cohen’s Kappa to account for the ordinal nature of the grading system ((1) slight bulging, (2) small herniated disc, (3) moderate herniation, (4) severe herniation). Interpretations of kappa values were based on the Landis and Koch benchmark scale.

The inter-rater reliability between Rater A and Rater B demonstrated a weighted kappa of 0.77 (95% CI: 0.68–0.86), indicating substantial agreement. Moreover, the intra-rater reliability for Rater A, comparing scores at two different time points, yielded a weighted kappa of 0.84 (95% CI: 0.76–0.92).

In conclusion, the MRI herniation grading system demonstrated substantial to almost perfect reliability, supporting its use for standardized evaluation in both clinical and research settings.

## Appendix B. Linear Regression

**Table A1:** Linear regression models adjusted for gender, age and BMI comparing large versus small herniation size.

		NECK Trial (Surgical)			CASINO Trial (Surgical)			Surgical (Nerge)			CASINO (Conservative)			NECK + CASINO		
		B	95% CI	*p*	B	95% CI	*p*	B	95% CI	*p*	B	95% CI	*p*	B	95% CI	*p*
**NDI**	**Baseline**	0.53	−5.4; 6.9	0.868	0.10	−7.6; 7.8	0.979	1.43	−3.4; 6.3	0.559	2.55	−8.9; 14.0	0.653	2.23	−2.2; 6.7	0.327
	**1 yr FU**	−0.13	−7.6; 7.4	0.972	−4.49	−14.7; 5.7	0.376	0.27	−9.5; 6.1	0.927	1.74	−18.9; 22.3	0.863	−0.04	−5.8; 5.7	0.989
**VAS arm**	**Baseline**	−4.09	−12.8; 4.6	0.350	0.42	−13.0; 13.9	0.950	−2.49	−9.5; 4.5	0.480	−5.73	−23.3; 11.8	0.511	−2.78	−9.4; 3.8	0.408
	**1 yr FU**	3.47	−7.3; 14.3	0.525	−0.64	−14.8; 13.5	0.927	4.69	−3.7; 13.1	0.270	0.975	−16.0; 17.9	0.906	4.52	−2.8; 11.8	0.224
**VAS neck**	**Baseline**	−0.71	−11.6; 10.2	0.897	3.55	−12.9; 20.1	0.668	−0.06	−8.8; 8.6	0.989	−5.18	−24.2; 13.9	0.585	−0.65	−8.4; 7.1	0.868
	**1 yr FU**	−6.37	−17.0; 4.3	0.239	10.65	−6.3; 27.6	0.210	−0.32	−9.0; 8.3	0.941	5.86	−17.5; 29.2	0.610	0.531	−7.4; 8.4	0.894
**EQ VAS**	**Baseline**	2.64	−7.4; 12.6	0.601	1.15	−12.6; 14.9	0.867	1.48	−6.2; 9.2	0.705	−4.94	−19.4; 9.5	0.493	0.488	−6.2; 7.2	0.886
	**1 yr FU**	−3.55	−12.7; 6.0	0.442	−5.71	−17.3; 5.9	0.325	−5.45	−12.6; 1.7	0.131	−2.83	−24.6; 18.9	0.792	−3.12	−10.0; 3.8	0.374
